# The N-Terminus of the RNA Polymerase from Infectious Pancreatic Necrosis Virus Is the Determinant of Genome Attachment

**DOI:** 10.1371/journal.ppat.1002085

**Published:** 2011-06-23

**Authors:** Stephen C. Graham, L. Peter Sarin, Mohammad W. Bahar, Reg A. Myers, David I. Stuart, Dennis H. Bamford, Jonathan M. Grimes

**Affiliations:** 1 Division of Structural Biology, Wellcome Trust Centre for Human Genetics, University of Oxford, Oxford, United Kingdom; 2 Institute of Biotechnology and Department of Biosciences, University of Helsinki, Biocenter 2, Helsinki, Finland; University of Kentucky, United States of America

## Abstract

The RNA-dependent RNA polymerase VP1 of infectious pancreatic necrosis virus (IPNV) is a single polypeptide responsible for both viral RNA transcription and genome replication. Sequence analysis identifies IPNV VP1 as having an unusual active site topology. We have purified, crystallized and solved the structure of IPNV VP1 to 2.3 Å resolution in its *apo* form and at 2.2 Å resolution bound to the catalytically-activating metal magnesium. We find that recombinantly-expressed VP1 is highly active for RNA transcription and replication, yielding both free and polymerase-attached RNA products. IPNV VP1 also possesses terminal (deoxy)nucleotide transferase, RNA-dependent DNA polymerase (reverse transcriptase) and template-independent self-guanylylation activity. The N-terminus of VP1 interacts with the active-site cleft and we show that the N-terminal serine residue is required for formation of covalent RNA∶polymerase complexes, providing a mechanism for the genesis of viral genome∶polymerase complexes observed *in vivo*.

## Author Summary

Infectious pancreatic necrosis virus (IPNV) is highly contagious and causes severe disease in fish. As a result of intensive rearing conditions it has become a serious problem for the salmon and trout farming industries. IPNV, like many other viruses, replicates its genome using a protein (a ‘polymerase’) that is itself encoded by the viral genome. Unusually, in infectious IPNV particles the polymerase is found chemically linked to the viral genome. We have determined the atomic structure of IPNV polymerase using X-ray crystallography, revealing some significant differences in the fold of the protein chain compared to other well-characterized viral polymerases. By mutating an amino acid residue at the beginning of the protein we show how the chemical linkage to the viral genome can be disrupted. This provides an elegant mechanism for the attachment of the viral genome to the polymerase observed *in vivo*.

## Introduction

Virus-encoded RNA-dependent RNA polymerases (RdRPs) are vital components in the life cycle of RNA viruses, being responsible for both genome replication and transcription. RdRPs from viruses that are considered unrelated [e.g. hepatitis C virus (family *Flaviviridae*), Foot-and-Mouth disease virus (family *Picornaviridae*) and bacteriophage Φ6 (family *Cystoviridae*)] share a similar structure and mechanism of catalysis and are therefore potential targets for generic antiviral drugs [1]. They all have the canonical “right-hand” polymerase fold with *palm*, *fingers* and *thumb* domains and contain a number of conserved sequence motifs (*A*–*G*), the conserved residues GDD within motif *C* being essential for catalytic activity [2], [3].

Members of the family *Birnaviridae* possess bi-segmented double-stranded RNA (dsRNA) genomes and form virions with non-enveloped single-shelled icosahedral capsids [4]. Infectious pancreatic necrosis virus (IPNV), which infects salmonids, and infectious bursal disease virus (IBDV), a poultry virus, are pathogens of significant economic importance and are the best characterized members of this virus family [5], [6]. From sequence analysis it was proposed that the RdRPs of *Birnaviridae* possess a non-canonical active site where the polymerase sequence motifs are re-ordered *C*-*A*-*B*, motif *C* containing ADN in place of the conserved GDD catalytic residues [3]. The structure of the RdRP VP1 from IBDV confirmed this hypothesis [7], revealing that the polymerase catalytic site was spatially similar to canonical viral RdRPs despite the different connectivity of the catalytic motifs. However, it remained unclear how substrate and metal ions bound at the active site during catalysis.

An additional unusual feature of birnavirus RdRPs is their ability to self-guanylylate: auto-catalyzing the covalent addition of a GMP moiety to a serine residue of the polymerase in a template-independent manner to form VP1pG [8], [9], [10]. This self-guanylylation has been presumed to be involved in priming the initiation of replication [11]. VP1 is observed in the virion as two forms, free polymerase and covalently attached to the 5′ ends of the segments of the viral RNA genome [12], and recent studies have shown that recombinant IBDV VP1 produces both free and protein-attached RNA products [9]. The exact site of self-guanylylation is unclear. Biochemical characterization mapped the self-guanylylation to residue S163 of IPNV VP1 [10], but no evidence for guanylylation was observed at an equivalent site in structures of the IBDV enzyme [7], [13]. Recent studies of IBDV VP1 suggest that the self-guanylylation reaction occurs in the first 175 residues of the enzyme at a locus distinct from the polymerase catalytic site [9].

We have cloned, expressed and solved the structure of the RdRP VP1 from IPNV in both its *apo* form and bound to a catalytic metal (magnesium). We show that IPNV VP1 possesses significant terminal (deoxy)nucleotide transferase and reverse transcriptase activity in addition to its RNA replication/transcription activity. Unexpectedly our structures reveal that the first 30 residues of VP1, not observed in the structure of IBDV VP1, bind at the active site of the enzyme. Mutation of the N-terminal serine residue of the mature protein to alanine abolishes its ability to form covalent RNA∶polymerase complexes, suggesting a mechanism of VP1∶genome attachment *in vivo*.

## Results

### The structure of C-terminally truncated (ΔC55) IPNV VP1

Recombinantly expressed IPNV VP1 carrying a C-terminal hexahistidine affinity tag is predominantly monomeric in solution as determined by analytical gel filtration and multi-angle light scattering (data not shown). Crystals of full-length VP1 with the C-terminal hexahistidine tag removed diffracted to 3.8 Å resolution but proved refractory to further optimization. A second construct lacking the C-terminal 55 residues (ΔC55) was therefore cloned, expressed, purified and crystallised. These crystals diffracted well and the structure was solved by molecular replacement using the structure of IBDV VP1 [7] as a search model. The final structure contains 5 molecules in the asymmetric unit and has been refined to 2.3 Å resolution with residuals *R* = 0.166, *R*_free_ = 0.187 (Table S1). The stereochemical quality of the structure is excellent, with 3756 of 3835 residues (98%) occupying favored regions of the Ramachandran plot (Table S1).

**Figure 1:**
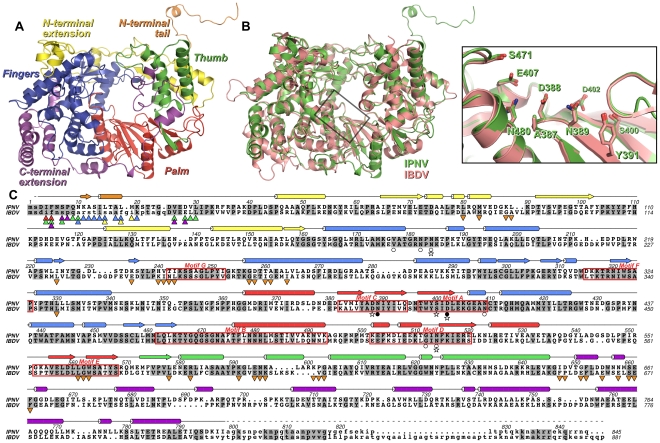
Structure of IPNV VP1. (A) Cartoon representation of the structure of IPNV ΔC55 VP1, colored to show the canonical RdRP *fingers* (blue), *thumb* (green) and *palm* (red) domains, the N-terminal (yellow) and C-terminal (magenta) extensions to the canonical RdRP fold and the N-terminal tail (orange). (B) Superposition of VP1 from IPNV (green) and IBDV (pink; PDB ID 2PGG). The inset shows the superposed active sites, for clarity only selected side chains are shown. (C) Structure-based sequence alignment of IPNV and IBDV VP1. IPNV secondary structure is shown above the sequences, colored as in (A). Conserved residues are highlighted in grey, the characteristic polymerase motifs are boxed and residues not observed in the structures are in lower case. Triangles denote residues of the N-terminal tail (▴) or polymerase domain (▾) that interact with each other, the color of the triangle corresponding to the region of VP1 with which this residue interacts (colors being as in (A)). Stars denote residues mutated in this study, filled circles represent Mg^2+^-binding residues and empty circles denote K^+^-binding residues.

The structure of ΔC55 IPNV VP1 comprises the polymerase domain (residues 31–790), an ordered helix of the N-terminus (residues 11–19; see below) and two residues of the C-terminal affinity tag. The IPNV polymerase closely resembles IBDV VP1, with an r.m.s. deviation in Cα positions of 1.2 Å over 678 residues (Figure 1). The structure of the core polymerase, comprising the *fingers*, *palm* and *thumb* domains, is especially well conserved (1.0 Å Cα r.m.s. deviation over 463 residues), with significant structural rearrangements occurring only at the tips or back of the *fingers* and *thumb*, distal to the catalytic *palm* domain (Figure 1). The structure of IPNV VP1 confirms the previously-identified rearrangement of the catalytic *palm* domain [3], [7], with the non-canonical ADN catalytic motif (residues 387–389) lying at the apex of a β-hairpin in the active site (Figure 1). As in IBDV VP1, extensive N- and C-terminal extensions wrap around the core *fingers*, *palm* and *thumb* domains (Figure 1). The sequence and structure of the N-terminal extension is largely conserved, differences occurring only in the conformation of the loop between residues 82–91. The general conformation of the C-terminal extension is similar to IBDV, passing from the inner-base of the *thumb* to the back of the *fingers* via the outer edge of the *palm*. However, many of the surface-exposed loops adopt different conformations and the helix-turn-helix between residues 736–779 is rotated by approximately 50° relative to the equivalent region of IBDV VP1.

**Figure 2:**
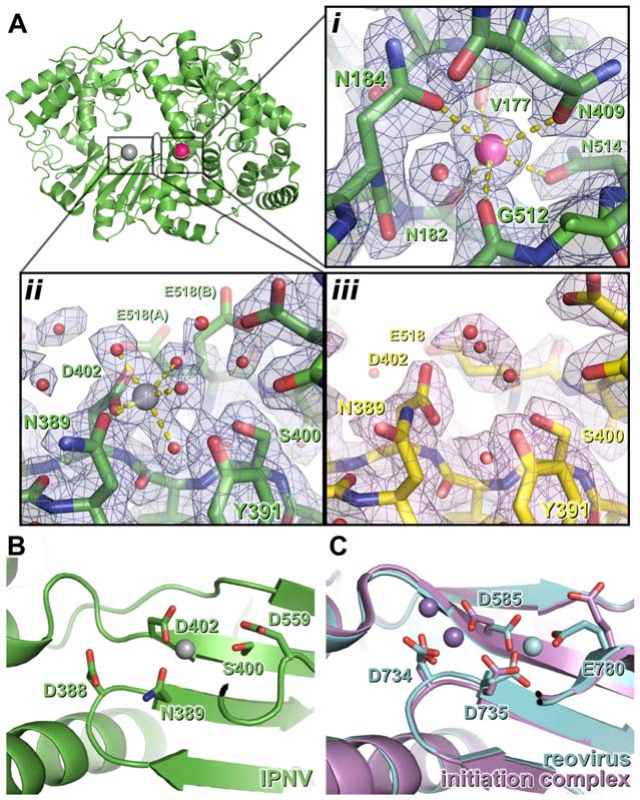
Metal binding of IPNV VP1. (A) The structure of IPNV ΔC55 VP1 soaked with Mg^2+^ is shown in cartoon representation, rotated by approximately 180° around a vertical axis relative to the orientation in Figure 1. Mg^2+^ and K^+^ ions are shown as oversized grey and pink spheres, respectively. (*i*) K^+^ binding site in 2*F*_O_-*F*_C_ electron density calculated from the final refined model (blue, 1.5 σ). (*ii*) Mg^2+^ binding site, electron density is as in (i). (*iii*) The active site of *apo* ΔC55 VP1 in 2*F*_O_-*F*_C_ electron density calculated from the final refined model (magenta, 1.5 σ). (B) Mg^2+^ (grey) binds away from the ‘tip’ of the motif *C* catalytic β hairpin at the active site of IPNV VP1. View is rotated by approximately 45° around the horizontal axis compared with (A). (C) Binding of the Mn^2+^ at the active site of unliganded reovirus RdRP (cyan; PBB ID 1MWH) and of a reovirus RdRP initiation complex (purple; PBB ID 1N1H). View is as in (B). For clarity, in (B) and (C) only selected side chains are shown and NTPs are omitted.

Strong electron density was observed near the outer edge of the *palm* domain, between the side chains of N184, N409 and N514 and the backbone carbonyl oxygen atoms of V177, N182 and G512 (Figure 2). This feature, some 15 Å from the putative active site, was modeled as a K^+^ ion based on the strength of the electron density, the ligand-to-K^+^ distances of 2.6–2.8 Å [14] and the *hard* nature of the six ligands.

### The structure of Mg^2+^-bound ΔC55 VP1

To characterize binding of catalytic metals at the active site of the enzyme, crystals of ΔC55 VP1 were soaked in reservoir solution supplemented with 50 mM MgCl_2_ and 10 µM GTP and lacking citrate (which chelated metals, frustrating soaking experiments). The Mg-bound ΔC55 VP1 structure was refined to 2.2 Å resolution with residuals *R* = 0.161, *R*_free_ = 0.181 (Table S1). A single Mg^2+^ ion was observed bound at the active site, coordinated by four solvent molecules and the side chain oxygen atoms of N389 and D402 in an octahedral geometry (Figure 2).

The coordination geometry of the bound K^+^ ion does not change upon addition of Mg^2+^, suggesting that Mg^2+^ does not replace K^+^ in this position. As in the structure of IBDV VP1 soaked with Mg^2+^ and GTP [13], no significant structural rearrangements were observed between the structures of *apo* and Mg-bound ΔC55 VP1 and no electron density for a bound GTP molecule was observed.

### The N-terminus of VP1 interacts with the active site

Additional electron density resembling a protein helix was observed in the active site cleft of both *apo* and Mg-bound ΔC55 VP1. Based on the presence of strong density suggesting a sulfur-containing methionine side chain, this helix was tentatively assigned as residues 11–19 from the otherwise-disordered N-terminal tail of the protein. As the 48 Å separating the final residue of the helix (M19) and the first residue of the polymerase domain (I31) is too great to be spanned by the intervening 10 residues, the N-terminal helix presumably derives from an adjacent polymerase domain in the crystal.

**Figure 3:**
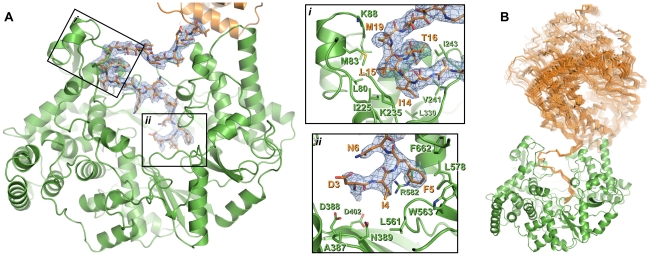
Interaction of the N-terminal tail with the polymerase domain of VP1. (A) The polymerase domain of VP1 is shown in cartoon representation (green), the N-terminal tail that interacts with the active site of the polymerase domain is shown as sticks (orange) and the polymerase domain from which this N-terminal tail extends is shown in cartoon representation (orange). 2*F*_O_-*F*_C_ electron density calculated from the final refined model is shown in blue (1.0 σ); for clarity density is shown only within a 2 Å radius of N-terminal tail residues. Insets (i) and (ii) show close-up views of the interaction between the N-terminal tail and the polymerase domain. For clarity side chains are shown only for polymerase domain residues that interact with the N-terminal tail or lie at the active site, and only selected residues are labeled. (B) Orientation of the polymerase domain from which the N-terminal tail extends (orange Cα trace) relative to the polymerase domain to which the N-terminal tail binds (green cartoon). Sixteen independent views of the interacting polymerase domain (from three different crystal forms) are shown, all having been superposed upon residues 11–19 of a reference interaction.

The identity of the N-terminal helix was confirmed by solving the structure of ΔC55 VP1 in an alternate crystal form at 3.0 Å resolution with residuals *R* = 0.188, *R*_free_ = 0.216 (‘large unit cell’, Table S1). Clear electron density connects the helix with residue 31 of an adjacent molecule in the crystal in 7 of the 8 molecules present in the asymmetric unit (Figure 3, Figure S1). In addition, residues 3–10 are seen in this structure extending toward the catalytic active site (Figure 3, Figure S1). As in the high-resolution structures, the helix formed by residues 13–19 lies in a hydrophobic pocket within the active site cleft formed by loops of the *fingers* domain and the N-terminal extension (Figure 3, Figure S1). Residues 11–12 form a short isolated β strand which interacts with residue 241 of the *fingers* domain, while residue 10 spans the two sides of the active site cleft. Residues 7–10 interact with a loop between residues 594–598 of the *thumb*, this loop being partly mobile and modeled as having two conformations in the refined structure. Residues 3–6 interact with the *thumb*, the *palm* and the C-terminal extension, the side chain of F5 being buried in a hydrophobic pocket between the side chains of residues W563, L578, R582 and F662 (Figure 3). While the conformation of S2 (the first residue of mature VP1 assuming removal of the initiator methionine by methionine aminopeptidase) could not be assigned unambiguously, it will clearly lie extremely close to the catalytic residues of the active site (Figure 3).

### The structure of full-length VP1

The structure of full-length IPNV VP1 was solved using ΔC55 VP1 as a search model and refined to 3.8 Å resolution with residuals *R* = 0.186, *R*_free_ = 0.213 (Table S1). As for ΔC55 VP1 in the large unit cell, electron density for residues 3–19 was present in the active site. Some residual electron density could be observed linking residue 19 to the first residue (E27) of an adjacent polymerase molecule, but it was not sufficiently well-resolved to allow modeling of the intervening residues. Despite being crystallized in the presence of Mn^2+^ and ATP, no metal ions or nucleotides were observed at the active site. While additional electron density was seen connected to the C-terminus of the molecular replacement search model in three of the four copies of VP1 present in the asymmetric unit only eight additional residues (791–798) could be placed, suggesting that residues 799–845 are not ordered. Surprisingly the C-terminus of the fourth molecule in the asymmetric unit (from residue 687 onwards) is significantly refolded compared to the other IPNV VP1 molecules (Figure S2) and IBDV VP1. The strand that runs along the side of the *palm* domain is shifted ‘upward’ to lie at the outer rim of the active site cleft. Neither the helix that lay between the base of the *palm* and *fingers* (residues 706–716), the three-helix bundle (residues 724–781) nor the intervening loop region are evident; instead the polypeptide has flipped up and projects away from the back of the *fingers*. A continuous stretch of density returning from this projection covers the back of the *fingers* and extends to cover the back base of the *fingers* and the N-terminal extension, but given the low resolution of the data we were unable to include this region in the final refined model.

### Biochemical characterization of IPNV VP1

**Figure 4:**
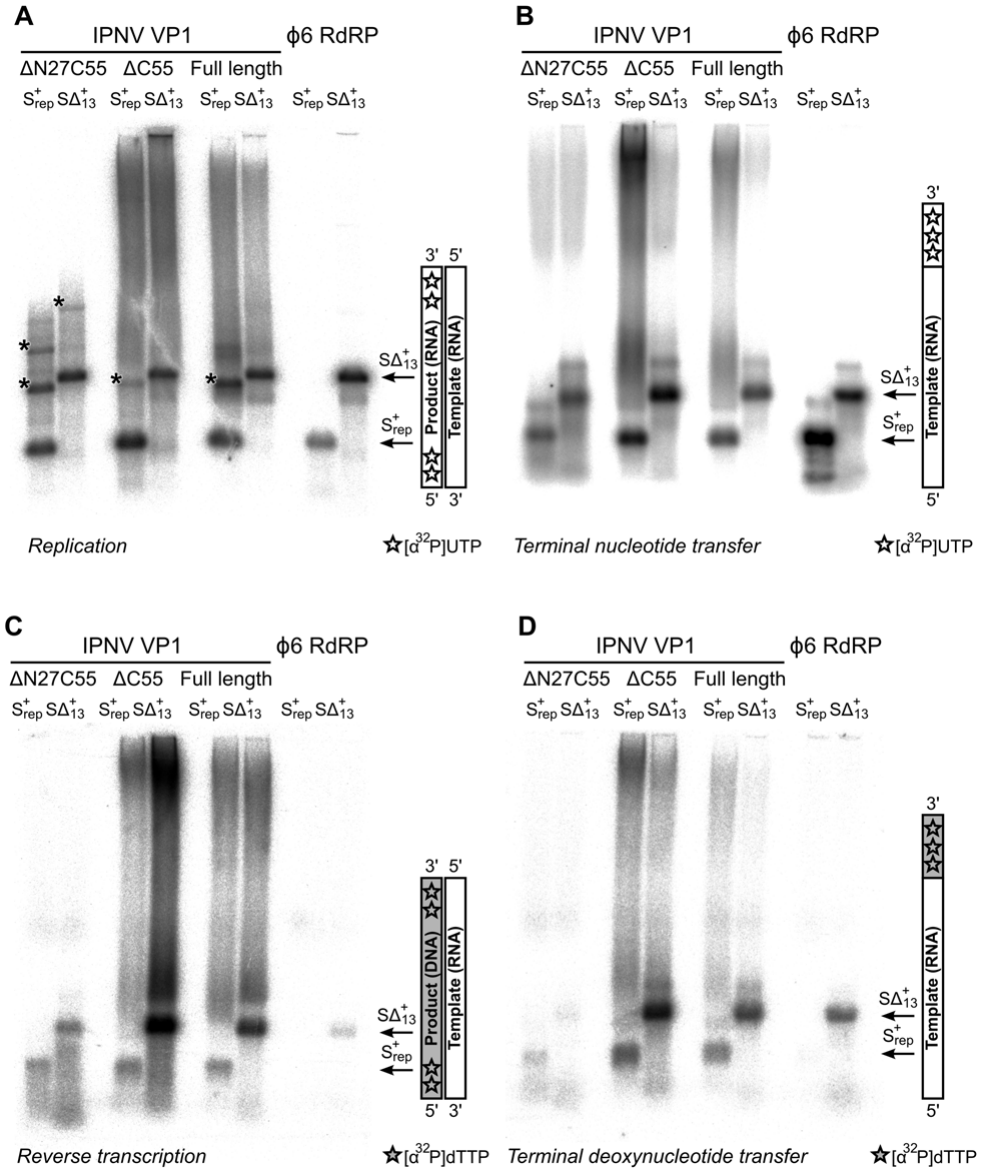
Catalytic activities of recombinant IPNV VP1. Autoradiography following agarose gel electrophoresis of [α^32^P]-UTP (A and B) or [α^32^P]-dTTP (C and D) labeled (A) *de novo* replication, (B) terminal nucleotide transfer, (C) reverse transcription and (D) terminal deoxynucleotide transfer reactions catalyzed by ΔN27C55, ΔC55 and full-length VP1 or the Φ6 RdRP. Note the high molecular weight smear present in reactions catalyzed by full-length or ΔC55 VP1, absent in reactions catalyzed by ΔN27C55 VP1, and the presence of discrete larger reaction products in (A) consistent with concatenation of product RNA chains (marked with *).

Full-length VP1, the ΔC55 construct used to generate well-diffracting crystals, and an N- and C-terminally truncated construct (ΔN27C55) that represents the well-folded VP1 polymerase domain (residues 28–790) were biochemically characterized. All recombinant VP1 constructs are capable of initiating *de novo* synthesis with non-specific single-stranded (ss) and double-stranded (ds) RNA templates, but not with ssDNA or dsDNA molecules (Figure 4, Table 1 and data not shown). VP1-catalysed polymerization proceeds readily in the presence of divalent cations and nucleotides (NTPs or dNTPs) and produces full-length double-stranded products (dsRNA or RNA/DNA hybrids, see below) of all tested ssRNA templates. Full-length and truncated VP1 are less productive than the highly-active RdRP from bacteriophage Φ6, which was used for comparison (Figure 4, Table 1). VP1 can perform *in vitro* semi-conservative transcription with dsRNA templates, albeit less efficiently than replication (Table 1).

**Table 1:** Biochemical characterization of the IPNV VP1 polymerases.

Activity	Template^a^	VP1	VP1 ΔC55	VP1 ΔN27C55	Φ6 RdRP
**Replication** b	sΔ^+^_13_	71%	98%	72%	100%
	s^+^_13_	5%	6%	21%	100%
**Elongation rate**	sΔ^+^_13_	1.2 nt/s	1.2 nt/s	0.7 nt/s	30 nt/s
**Transcription** b	Φ6 genome	5%	25%	5%	100%
**Back-priming** c	sΔ^+^_13_	61%	30%	27%	47%
	sΔ^+^_HP_	78%	42%	27%	73%
**RNA∶polymerase complex formation** d	s^+^_rep_	35%	9.3%	nd	nd
**TNTase** b	sΔ^+^_13_	0.1%	2.5%	14%	100%
**TdNTase** b	sΔ^+^_13_	60%	343%	2.5%	100%
**Reverse transcriptase** e	sΔ^+^_13_	100%	446%	19%	nd
**Terminal nucleotide Preference** f	sΔ^+^_A_	76%	77%	57%	25%
	sΔ^+^_C_	100%	100%	100%	100%
	sΔ^+^_G_	96%	98%	86%	30%
	sΔ^+^_U_	61%	69%	37%	33%

a sΔ^+^_13_: 723 nt ssRNA; s^+^_13_: 2961 nt ssRNA; Φ6 genome, 6374 bp, 4063 bp and 2948 bp dsRNA; sΔ^+^_HP_: 724 nt ssRNA with 3′ hairpin loop; s^+^_rep_: 300 bp ssRNA; sΔ^+^_A,C,G,U_: 711 nt ssRNA with 3′ adenosine, cytidine, guanosine and uridine nucleotides (respectively).

b Normalized against Φ6 RdRP activity.

c Percent of total dsRNA synthesis.

d Percent of total RNA product found covalently bound to the polymerase, nd = none detected.

e Normalized against VP1 activity, nd = none detected.

f Normalized against highest value.

VP1 is devoid of activity in the absence of divalent cations, whereas addition of Mg^2+^ or Mn^2+^ at concentrations exceeding 2 mM stimulates RNA synthesis (Figure S3). Adding Ca^2+^ as the sole divalent cation to the reaction mixture does not induce catalysis (data not shown) and supplementing the Mg^2+^- or Mn^2+^-containing reaction mixtures with Ca^2+^ completely inhibits polymerization (data not shown). The addition of Zn^2+^ yields low levels of RNA synthesis at a concentration of 2 mM or less, whereas higher concentrations efficiently inhibit catalysis (data not shown). Supplementing reaction mixtures with K^+^ does not stimulate RNA synthesis, and K^+^ on its own is unable to induce catalysis (data not shown). Interestingly, adding both Mg^2+^ and Mn^2+^ to the reaction mixture at their respective optimal concentration has an adverse effect on RNA synthesis and RNA polymerization is most efficient when using half the optimal concentration of both ions (5 mM Mg^2+^ and 1 mM Mn^2+^; data not shown).

VP1 is not able to initiate primer-dependent RNA synthesis but displaces various complementary RNA oligonucleotides annealed to ssRNA templates (Figure S4) despite poor *in vitro* transcription activity (Table 1). Synthesis of a significant proportion of RNA strands is initiated by means of back-priming (Table 1), as observed previously for IBDV VP1 [15]. IPNV VP1 shows only a slight preference for cytidine as the terminal template nucleotide (Table 1). The elongation rate of IPNV VP1 is slow when compared with the Φ6 RdRP (Table 1; Figure 4) and VP1 reaches a higher product yield with short (<500 nucleotide) ssRNA templates (Figure 4).

Based on structural data, we designed a set of mutant polymerases to verify the identity of the catalytic center. Single point mutations were introduced at the putative active site (D388N, D402N and S400A) and at the K^+^ binding site (N184S and N514H). The replication activity of all these mutant polymerases was below the detection limit of our assays (<5% of wild-type VP1 activity, data not shown).

### Terminal (deoxy)nucleotide transferase and reverse transcriptase activity

IPNV VP1 is capable of utilizing free NTPs or dNTPs for the template-independent addition of one or more nucleotides to the 3′ terminus of single- or double-stranded RNA, so-called terminal (deoxy)nucleotide transferase (TNTase and TdNTase) activity (Figure 4). The TNTase activity of VP1 is very weak compared to that of Φ6 RdRP, although this activity is significantly enhanced in the truncated VP1 constructs (Figure 4, Table 1). Somewhat surprisingly, ΔC55 VP1 has a 5-fold higher TdNTase activity than the full-length enzyme, surpassing the equivalent activity of the Φ6 RdRP by more than two-fold, while the TdNTase activity of ΔN27C55 VP1 is severely compromised (Table 1).

In addition to the TdNTase activity of IPNV VP1, the polymerase is capable of utilizing a ssRNA template and dNTPs for *de novo* RNA-directed DNA polymerization resulting in a RNA/DNA hybrid (Figure 4, Figure S5). This reverse transcriptase activity is less efficient than *in vitro* RNA replication when utilizing the same ssRNA template. Both full-length and ΔC55 VP1 yield RNA/DNA hybrids of various heterologous ssRNA templates, ΔC55 being roughly 5-fold more active than the full-length enzyme, while ΔN27C55 VP1 reverse transcriptase activity is extremely low (Table 1, Figure 4).

### Self-guanylylation and covalent RNA∶polymerase complex formation

**Figure 5:**
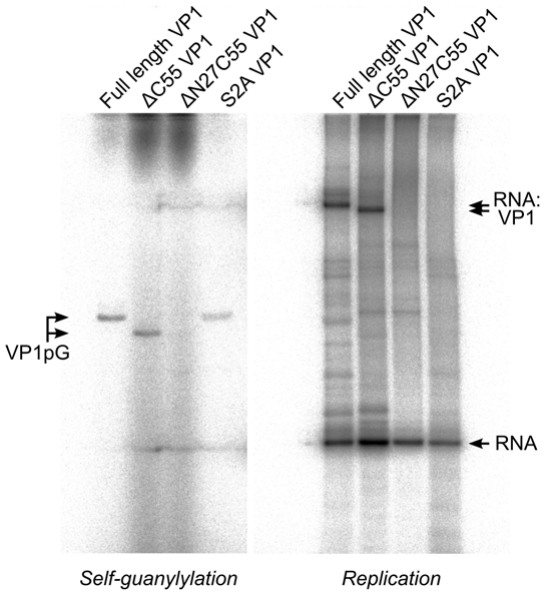
IPNV VP1 self-guanylylation and RNA∶polymerase complex formation. Autoradiography following denaturing SDS PAGE of IPNV VP1 self-guanylylation (left panel) and *de novo* RNA polymerization using s^+^_rep_ template (300 nt ssRNA, right panel) in the presence of [α^32^P]-GTP. Full-length and ΔC55 VP1 self-guanylylate to form covalent VP1pG complexes (left panel) and form covalent RNA∶polymerase complexes migrating at approximately 180–200 kDa (right panel). ΔN27C55 VP1 lacks both these activities, while an S2A mutant of the full-length enzyme retains self-guanylylation activity but lacks the ability to form covalent RNA∶polymerase complexes.

Upon incubation with [α^32^P]-GTP both full-length and ΔC55 VP1 acquire radio-labels that do not dissociate during denaturing gel electrophoresis (Figure 5), presumably because the proteins have covalently bound [α^32^P]-GMP to form VP1pG (so-called self-guanylylation activity) [8]. A mutant form of VP1 where the N-terminal serine residue of the mature protein has been mutated to alanine (S2A) maintains the ability to form VP1pG, while ΔN27C55 VP1 is not radio-labeled under denaturing conditions and therefore lacks self-guanylylation activity (Figure 5).

Replication of ssRNA by IPNV VP1 yields high molecular weight products in addition to the expected dsRNA, manifesting as ladders and smears of radioactivity on native agarose gel electrophoresis [8]. The formation of ladders is most marked when using short RNA templates and is consistent with the formation of ‘concatenated’ RNA products containing integral repeats of the template RNA (Figure 4A and data not shown). RNA concatenation activity is strongest for ΔN27C55 VP1, while formation of the larger heterogeneous smears is more pronounced for full-length and ΔC55 VP1 (Figure 4A). Following denaturing sodium dodecyl sulfate polyacrylamide gel electrophoresis (SDS PAGE; Figure 5) two major products are observed: free RNA product and RNA∶polymerase complexes that represent covalent association of VP1 with the nascent RNA [9]. However, while ΔN27C55 and S2A VP1 are capable of producing RNA product, they lack the ability to form covalent RNA∶polymerase complexes.

## Discussion

We have expressed and purified the IPNV RNA-dependent RNA polymerase VP1 and determined its structure to 2.2 Å resolution. The structure closely resembles that of IBDV VP1 (Figure 1), confirming that birnavirus polymerases constitute a family of RNA virus polymerases with a permuted motif *C* relative to other (canonical) viral single-polypeptide RdRPs [3], [7]. Recombinant IPNV VP1 is highly active, utilizing NTPs to catalyze the replication/transcription of ssRNA/dsRNA templates in a reaction that does not rely on added RNA primers (primer-independent or *de novo* initiation; Figure 4) in order to produce both free RNA product and RNA that remains covalently bound to the polymerase (Figure 5). VP1 is also capable of adding NTPs and dNTPs to the 3′ terminus of reaction products in a template-independent fashion (TNTase/TdNTase activity; Figure 4). Furthermore, it can utilize dNTPs and ssRNA templates in the absence of DNA primers to produce an RNA/DNA hybrid (reverse transcriptase activity; Figure 4, Figure S5). Mutations of residues D388, S400 and D402 abolish replication, transcription and TNTase/TdNTase activity, confirming that the catalytic active site lies within the *palm* domain. The spatial organization of the active site is similar to that of IBDV VP1 (Figure 1) and of canonical viral RdRPs such as the Φ6 RdRP [7], [9], [16]. Removal of the C-terminal 55 residues (residues 791–845) of VP1 enhances the replication, transcription and TNTase/TdNTase activities of the enzyme; however, as this region was not ordered in the structure of full-length VP1, its role in modulating activity remains unclear. Further removal of the N-terminal 27 amino acids yields an enzyme that is competent to replicate/transcribe RNA and has enhanced TNTase activity, but has a reduced ability to utilize dNTPs (Table 1) and lacks both self-guanylylation activity and the ability to covalently associate with RNA product (Figure 5). A VP1 mutant where the N-terminal serine of the mature enzyme is replaced with alanine (S2A) maintains RNA replication and self-guanylylation activity but does not form covalent RNA∶polymerase complexes (Figure 5).

### Metal binding at the VP1 catalytic site

IPNV VP1 requires metal ions for catalysis, optimal activity being obtained in the presence of 5 mM Mg^2+^ and 1 mM Mn^2+^ (Figure S3 and data not shown). However, when soaked with an excess of Mg^2+^ ions we observe only one Mg^2+^ ion at the active site, coordinated by the side chains of N389 and D402 (Figure 2). It was reported that three Mg^2+^ ions were bound in the 3.15 Å structure of Mg-bound IBDV VP1 [13], however none of these occupy the Mg^2+^ site we observe in IPNV VP1. We performed additional experiments, soaking ΔC55 IPNV VP1 crystals with up to 200 mM Mg^2+^ and 40 mM Mn^2+^, but these failed to reveal any additional bound Mg^2+^ (data not shown). In the light of these results and inspection of the IBDV electron density maps, we ascribe differences in the observed metal binding to difficulties in interpreting the low resolution electron density of the earlier IBDV VP1 structure.

In initiation or elongation complexes of canonical viral polymerases two catalytic metal ions (plus one structural metal in the case of Φ6 RdRP) are observed at the active site [16], [17], [18], [19]. In such complexes the catalytic metal ions bridge the conserved Asp residues of catalytic motifs *A* and *C*, lying above the tip of the β hairpin (formed by motif *C*), and the triphosphate group of the substrate nucleotide. In the structure of reovirus RdRP solved in the absence of substrate nucleotides a single Mn^2+^ ion is observed in the active site in a position corresponding to that we observe for the single Mg^2+^ of IPNV VP1 [18], being coordinated by the side chains of D735, D585 and E780 (equivalent to VP1 residues N389, D402 and half-way between D559 and S400, respectively; Figure 2). Upon formation of an initiation complex two Mn^2+^ ions bind the reovirus RdRP active site (Figure 2). It is therefore likely that in the presence of template and nucleotide substrates a second metal ion will bind at the active site of IPNV VP1 and the resulting initiation complex will contain two metal ions coordinated by D388, D402 and the nucleotide triphosphate moiety at the tip of the catalytic motif *C* β hairpin.

### Birnavirus polymerases possess a structural K^+^ ion

In addition to the Mg^2+^ ion bound at the catalytic active site, in all structures of IPNV VP1 we observe a K^+^ ion bound at the junction of the *palm* and *fingers* domains (Figure 2). We find that density consistent with a K^+^ ion is also present in previously published structures of IBDV VP1, although it was not identified as such at the time. The residues that interact with the K^+^ ion (N184, N409 and N514) are conserved across the *Birnaviridae* (Figure S6) and we propose that binding of this K^+^ ion is a general feature of birnavirus polymerases. The region at which the K^+^ ion binds is not structurally conserved in canonical RdRPs and the K^+^ ion is not analogous to ‘structural’ metal ions such as the divalent cation bound on top of the *palm* near the catalytic residues in the Φ6 RdRP [20]. Mutations N184S or N514H (both predicted to prevent K^+^ binding) abolish polymerase activity, consistent with the K^+^ ion performing a role in structuring the *palm* and maintaining its orientation relative to the *fingers*.

### VP1 reverse transcriptase activity

IPNV VP1 has significant reverse transcriptase activity, utilizing dNTPs and RNA to form DNA/RNA hybrids (Figure 4, Figure S5). VP1 RNA-dependent RNA polymerization and reverse transcriptase activities share a requirement for RNA (not DNA) template, are optimally active under similar conditions, and are both abolished by mutations to the active site residues D388, S400 and D402, consistent with the two activities sharing a common catalytic mechanism.

The molecular features of VP1 that allow it to utilize dNTPs in addition to NTPs are not obvious. Mutation of F155 to valine in Moloney murine lukemia virus reverse transcriptase removes its specificity for dNTPs [17], [21]. Birnavirus RdRPs have a conserved glutamic acid at the equivalent position (E407 in IPNV, Figure S6), while in other viral RdRPs this residue is aspartic acid [7]. It had been suggested that this aspartate residue might recognize substrate NTPs by forming a hydrogen bond with the NTP O2′ atom [22], but structures of Φ6 and Norwalk virus RdRP initiation complexes contradict this hypothesis [16], [19]. The presence of asparagine in catalytic motif *B* near the tip of the motif *C* hairpin is unable to explain the preference of viral RdRPs for NTPs over dNTPs [23], [24] since this residue is asparagine in IPNV VP1 (N480, Figure 1). It is also unlikely that serine in the loop preceding the motif *B* helix (IPNV S471) is crucial for (d)NTP selectivity [7], as serine or threonine at this position is generally conserved across RdRPs not competent for reverse transcriptase activity.

Removal of the N-terminal 27 residues of IPNV VP1 greatly diminishes the reverse transcriptase activity of the enzyme and ΔN27C55 VP1 has enhanced TNTase activity but severely attenuated TdNTase activity. Based on a structural alignment with the Φ6 RdRP we observe that the N-terminus of the polymerase domain is adjacent to the (d)NTP entry tunnel (Figure S7), suggesting that the N-terminal segment of VP1 plays a role in recruiting dNTPs for catalysis. Further work is required to understand the molecular features that define NTP versus dNTP selectivity in viral RdRPs.

### Flexibility of the VP1 C-terminus

Residues 688 onwards are completely refolded in one of the four molecules present in the full-length VP1 structure (Figure S2). The functional implications of this reorganization are not obvious. Models of a VP1 elongation complex based on superposition of the reovirus RdRP elongation complex [18] or of HIV reverse transcriptase in complex with a DNA template [17] require refolding of a small segment of the VP1 C-terminal extension, residues 654–670, in order to accommodate the nascent RNA duplex (not shown). It is possible that the purpose of the C-terminal extension is to rigidify the polymerase during initiation, but that this extension must peel away to allow the growing nucleotide chain to exit the active site. The structure of VP1 where residues 688 onwards are refolded might thus represent an intermediate unfolding step rather than a conformation of biological significance in its own right.

### IPNV VP1 self-guanylylation

Birnavirus RdRPs hydrolyze GTP to form a covalent VP1pG complex in a template-independent manner, so-called self-guanylylation [8]. We see no evidence of covalent GMP modification at S163, the IPNV VP1 residue previously identified as being guanylylated [10], nor at any other position in the structures presented here or in crystals of VP1 co-crystallized with GTP (data not shown). Removal of the N-terminal 27 residues abolishes VP1 self-guanylylation activity (ΔN27C55 VP1, Figure 5), consistent with recent studies of IBDV VP1 that identified the site of self-guanylylation as residing within the first 175 residues of the polymerase [9]. We observe that ΔN27C55 VP1 maintains significant replication and transcription activity, demonstrating that self-guanylylation is not required for general “protein priming” of RNA polymerase activity. Further, as S2A VP1 self-guanylylates but lacks the ability to form covalent RNA∶polymerase complexes, the self-guanylylation activity is not sufficient for covalent attachment of RNA product to the enzyme (see below). While it is therefore clear that the N-terminal 27 residues of VP1 are required for self-guanylylation, the precise site and mechanism of this activity remains enigmatic.

### Role of the N-terminus in RNA∶polymerase complex formation

In 16 of the 17 independent molecular views of the IPNV VP1 structure presented here the flexible N-terminal tail of an adjacent VP1 molecule in the crystalline lattice lies anchored in the active site cleft (Figure 3). The affinity of this self-interaction is obviously not high as VP1 behaves predominantly as a monomer in solution (data not shown). However, the high concentration of VP1 in crystal structures may well reflect the environment of the viral capsid more closely than biochemical assays performed in dilute solution.

There are two main sites of interaction between the N-terminal tail and the active site cleft of IPNV VP1: F5 binds in a pocket formed by residues from the *thumb* and *palm*, and a helix (S13–M19) plus the two residues that precede it (K11–A12) bind a hydrophobic pocket formed by the *fingers* and N-terminal extension. F5 and the residues with which it interacts (W563, L578, R582 and F662) are highly conserved across birnavirus RdRPs (Figure S6), the conformations of these residues being identical in IBDV and IPNV VP1. A hydrophobic helix-binding pocket is also evident in IBDV VP1. The edge of the pocket proximal to the active site (IPNV residues 238–242) is similarly positioned in IPNV and IBDV VP1. The loops which form the edges of the pocket distal from the active site (IPNV residues 86–91 and 227–234) are very poorly ordered in IBDV VP1 structures, indicating significant flexibility, and they would require only modest rearrangement to accommodate a bound N-terminal helix. In crystal structures of IBDV VP1 there is no evidence for the N-terminal tail binding the active site [13]; however, the molecular packing does not bring N-termini of adjacent molecules into close enough proximity to interact with the active site, precluding the interaction.

**Figure 6:**
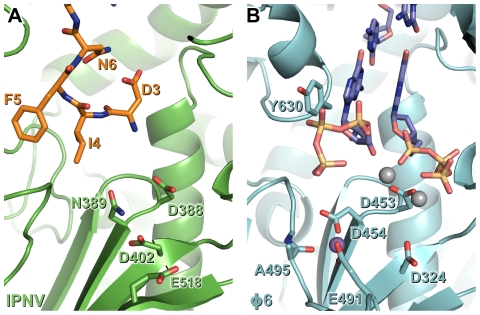
N-terminal tail binding at the active site of IPNV VP1 resembles the nascent daughter strand of a viral polymerase initiation complex. Mg^2+^ ions are shown in grey and Mn^2+^ ions in purple, and for clarity only selected side chains are shown. (A) IPNV VP1 (green) with residues from the N-terminal tail of an adjacent molecule in the crystal shown as sticks (orange). (B) Φ6 RdRP initiation complex (cyan; PDB ID 1HI0). The GTP molecules that will form the 5′ nucleotides of the nascent strand and the template 3′ nucleotides to which they Watson-Crick base pair are shown (blue).

The observed self-interaction brings the N-terminal residue of VP1 (S2, assuming removal of the initiator methionine) within approximately 5 Å of the catalytic site (Figure 3), and removal of the N-terminal tail or mutation of S2 to alanine abolishes the ability of VP1 to form covalent RNA∶polymerase complexes (Figure 5). Superposition of VP1 onto the initiation complex of the Φ6 RdRP shows the VP1 N-terminal tail to be located in approximately the same position as the 5′ nucleotide of a nascent RNA daughter strand (Figure 6). The N-terminal interaction is not required for “protein priming” since ΔN27C55 VP1 maintains significant replication activity (Figure 5). Instead, we propose that residue S2 at the N-terminus of VP1 represents the site of covalent attachment of RNA product to the polymerase. As there is no strong preference for templates with 3′ cytidine bases it is unlikely that the covalently-attached RNA is formed by extension of a self-guanylylation guanosine that Watson-Crick base pairs with template. Further, S2A VP1 is incapable of forming covalent RNA∶polymerase complexes despite maintaining self-guanylylation activity. This suggests that nascent RNA is directly ligated to S2, independently of initiation or of any self-guanylylation activity. Such a hypothesis, based on the observed interaction of the VP1 N-terminus with the active site and requirement for an N-terminal serine to generate covalent RNA∶polymerase complexes, provides an elegant molecular mechanism for the birnavirus VP1∶genome association observed *in vivo*.

## Materials and Methods

### Cloning, expression and purification of IPNV VP1

A pET-21a(+)–derived plasmid encoding full-length VP1 from IPNV strain Jasper (UniProt ID P22173) plus a C-terminal VEH_6_ tag [10] was the generous gift of Dr P. Dobos (University of Guelph). VP1 constructs lacking the C-terminal 55 residues (ΔC55) and N-terminal 27 plus C-terminal 55 residues (ΔN27C55 VP1) were cloned as described in Text S1. Site-directed mutagenesis of full-length IPNV VP1 was performed using the QuikChange site-directed mutagenesis kit (Stratagene). Full-length, ΔC55, ΔN27C55 and mutant VP1 were expressed and purified by Ni-immobilization affinity and gel-filtration chromatography using standard protocols (see Text S1). Pure VP1 was used immediately for crystallization or stored at −20°C in 50% v/v glycerol for kinetic analysis (conditions under which the enzyme remained stable and active for several months).

### Crystallization and data collection

Crystals of ΔC55 VP1 were grown in sitting drops containing 200 nL protein (5.8–6.8 mg/mL) and 100 nL reservoir solution (20–18% w/v PEG3350, 0.10–0.09 M bis-Tris propane pH 7.5, 0.2–0.18 M sodium citrate) equilibrated against 95 µL reservoirs at 20.5°C. Crystals were cryoprotected by soaking in mother-liquor supplemented with 20–25% glycerol for 1–10 min. Crystals of Mg-soaked ΔC55 VP1 were prepared by diluting the mother-liquor with ∼0.5 µL 20% w/v PEG3350, 0.1 M bis-Tris propane pH 8.25 and 20% v/v glycerol, transferring the crystals to a fresh drop containing 20% w/v PEG3350, 0.1 M bis-Tris propane pH 8.25, 20% v/v glycerol, 50 mM MgCl_2_ and 10 µM GTP, and incubating for 10 min. Crystals of full-length VP1 were grown in sitting drops containing 100 nL protein (5.3 mg/mL) plus 5 mM MnCl_2_ and 100 nL reservoir solution (20% w/v PEG3350, 0.1 M bis-Tris propane pH 7.5, 0.2 M sodium citrate, 20 mM ATP, 5% v/v MPD, 10 mM NaOH) equilibrated against 95 µL reservoirs at 20.5°C. All crystals were dipped into perfluoropolyether oil (PFO-X125/03, Lancester Synthesis) prior to cryocooling in a 100 K stream of N_2_ gas. Initial diffraction data collected on Diamond beam line I24 guided crystal optimization, from which diffraction data were recorded at Diamond beam line I02 (ΔC55 VP1) or ESRF beam line ID14-2 (Full-length VP1).

### Structure solution and refinement

Diffraction data were processed using MOSFLM [25] and SCALA [26] as implemented by xia2 [27]. Data processing statistics are shown in Table S1. The structure of Mg-soaked ΔC55 VP1 was solved by molecular replacement with PHASER [28] using the structure of IBDV VP1, PDB ID 2PGG [7], as a starting model. The structures of *apo* ΔC55 VP1, *apo* ΔC55 VP1 in an alternate (large) unit cell and of full-length VP1 were solved by molecular replacement using the polymerase domain of Mg-soaked ΔC55 VP1 (residues 31–792) as a starting model.

Manual building was performed using COOT [29] and structures were refined using BUSTER-TNT [30] in consultation with the MolProbity web server [31]. Non-crystallographic local structure similarity restraints [32] were used during the refinement of all structures. For full-length VP1 and *apo* ΔC55 VP1 in the large unit cell local structure similarity restraints [32] were also used to deter deviation of the models from the high-resolution *apo* ΔC55 VP1 structure unless compelling evidence for a difference was present in the electron density. As the test sets for all structures were chosen randomly (*apo* and Mg-bound ΔC55 VP1 sharing the same random set) the presence of non-crystallographic symmetry may artificially lower the value of *R*_free_ by a small amount but will not render the metric invalid [33]. Structural superpositions were performed using SSM [34] and Theseus [35]. Molecular graphics were prepared using PyMOL (DeLano Scientific) and sequence alignments with ALINE [36]. Structure factors and final refined coordinates have been deposited in the PDB with accession codes 2yi8 (*apo* ΔC55 VP1), 2yi9 (Mg-bound ΔC55 VP1), 2yia (*apo* ΔC55 VP1, large unit cell) and 2yib (full-length VP1).

### Biochemical analyses

The RdRP from bacteriophage Φ6 was expressed and purified as described previously [37] and RNA templates were generated as described in Text S1. Please refer to Table S2 for an overview of the RNA templates. The reaction conditions for RNA polymerization by IPNV VP1 were optimized using the 723 nucleotide (nt) ssRNA sΔ^+^_13_ template (Figure S3) and found to be similar to the ‘standard’ Φ6 RdRP reaction conditions (50 mM HEPES-KOH pH 7.5, 20 mM NH_4_Ac, 6% w/v PEG 4000, 5 mM MgCl_2_, 1 mM MnCl_2_, 0.1 mM EDTA, 0.1% v/v Triton X-100) [38]; these conditions were hence used for all subsequent experiments unless otherwise noted. Under optimized reaction conditions all VP1-catalysed reactions except reverse transcription were readily observable by ethidium bromide staining of agarose gel electrophoresis, indicating a high level of polymerase activity. Replication (ssRNA template) and transcription (dsRNA template) assays were carried out in the presence of 1 mM ATP and GTP and 0.2 mM CTP and UTP supplemented with labeled [α^32^P]-UTP (replication, transcription) or [α^32^P]-GTP (replication). TNTase, TdNTase and self-guanylylation assays contained only labeled nucleotides of [α^32^P]-UTP (TNTase), [α^32^P]-dTTP (TdNTase) or [α^32^P]-GTP (self-guanylylation). Reverse transcription was performed with 1 mM dATP and dGTP and 0.2 mM dCTP supplemented with 0.3 µM (0.1 mCi/mL) labeled [α^32^P]-dTTP. After 2 h incubation, reactions were stopped by the addition of 2× or 10× loading buffer [39], [40] and analysis of the reaction products was performed using 0.8% w/v agarose gel (TBE) electrophoresis [40]. [α^32^P]-GTP labeled self-guanylylation and replication assays were terminated with 7× SB loading buffer [40], boiled for 3 min and analyzed in 8% SDS polyacrylamide gels. Oligonucleotide displacement was assayed as previously described [41]. Signals were collected by autoradiography on BAS1500 image plates (Fujifilm), which were scanned using a Fuji BAS-1500 phosphorimager (Fujifilm). Digital image analysis (densitometry) was performed using AIDA Image Analyzer software (version 3.44; Raytest Isotopenmeβgeräte), measuring the band intensities in 1D Evaluation mode using Lane and Peak Determination.

## Supporting Information

Figure S1 **Binding of N-terminal tail to the VP1 active site cleft.** The N-terminal tail bound in the active site clefts of (A) Mg-bound ΔC55 VP1, and (B) ΔC55 VP1 (‘large unit cell’). Unbiased *F*_O_-*F*_C_ electron density, calculated before the N-terminal tail was added to the models, is shown in green (3.0 σ). The final refined structures of the polymerase domain and N-terminal tail are shown as a cartoon (white) and sticks (carbon atoms orange), respectively. In (B), the polymerase domain from which the N-terminal tail arises is shown as a cartoon (orange).
(TIFF)

Figure S2 **Reorganization of the C-terminal extension of full-length VP1.** (A) Full-length VP1 with the C-terminal extension in the commonly observed conformation (chains A–C) is shown as a white (residues 3–687) and magenta (residues 688–799) cartoon. 2*F*_O_-*F*_C_ electron density calculated from the final refined model is shown in blue (1.0 σ); for clarity density is shown only within a 2 Å radius of residues 688–799. (B) Full-length VP1 where the C-terminal extension is refolded (chain D) is shown as a white cartoon (residues 3–687) and a magenta Cα trace (residues 688 onwards). Electron density is as in (A), shown within a 3 Å radius of the Cα trace. Given the low resolution of the diffraction data we were unable to reliably build a main chain into the density and dock the sequence from residue 688 onwards in chain D. We have therefore excluded this region of the structure from the refinement, the deposited coordinates for this segment having zero occupancy to indicate this fact.
(TIFF)

Figure S3 **Biochemical characterization of IPNV VP1.** Screening the optimal reaction conditions for the IPNV VP1 in terms of (A) Mn^2+^ concentration, (B) Mg^2+^ concentration, (C) temperature, (D) ammonium acetate (NH_4_Ac) concentration, and (E) pH. Results are normalized against the highest attained polymerization activity for each RdRP in each experiment. Two ssRNA templates of different lengths (sΔ^+^_13_, 723 nt, and s^+^_13_, 2961 nt) were used. Interestingly the optimal temperature for RNA polymerization and final RNA yield obtained depends on the length of the ssRNA template. VP1 efficiently processes the short template (sΔ^+^_13_) at 37°C, whereas when a long template (s^+^_13_) is applied the RdRP is most active at 45–50°C. Higher reaction temperatures lead to increased degradation of the ssRNA template and a subsequent drop in RNA synthesis. The product yield with short ssRNA templates is much higher than with long, although in all cases the yield is sufficiently high to be detected by ethidium bromide (EtBr) staining following agarose gel electrophoresis (not shown).
(TIFF)

Figure S4 **Oligonucleotide displacement assay.** (A) Schematic representation of the secondary structure at the 3′ terminus of sΔ^+^ (upper panel) and of partially double-stranded sΔ^+^ RNA hybrids with 10 nucleotide (nt) and 20 nt 3′ overhangs (lower panels). The blue and red lines (lower panel) indicate the position at which the complementary RNA oligonucleotide anneals. (B) The RNA synthesis activity of full-length VP1 using sΔ^+^ ssRNA hybridized to short RNA oligonucleotides in order to produce RNA hybrids with different 3′ overhangs (0–575 nt) as template. The results have been normalized against unhybridized ssRNA sΔ^+^_13_ template (Native). The pre-initiation behavior of the VP1 polymerase is similar to that described for the Φ6 RdRP [41]. As with the Φ6 polymerase, RNA hybrids with single-stranded 3′ terminal overhangs of 0–2 nt induce very little RNA synthesis, whereas efficient RNA synthesis occurs using templates with 3′ overhangs of 3 nt or longer. Interestingly, the RNA hybrid with a 20 nt 3′ overhang stimulates approximately 2.5-fold higher RNA production activity than the unhybridized ssRNA sΔ^+^_13_ template.
(TIFF)

Figure S5 ***De novo*** **reverse transcription activity of wild-type VP1.** Left panel: Agarose gel electrophoresis and autoradiography of VP1 catalyzed primer independent reverse transcription. sΔ^+^_13_ ssRNA templated reverse transcription in the presence of [α32P]-dTTP leads to the formation of a labeled RNA/DNA hybrid (lane 1). Treatment with a mixture of DNases (lane 2) completely removes the signal. RNase treatment (lane 3) does not affect the sample intensity, indicating that the label becomes incorporated into the newly synthesized complementary DNA strand. A combination of RNases and DNases removes the signal completely (lane 4). Right panel: Agarose gel electrophoresis and autoradiography of PCR amplification of the labeled cDNA using Phusion HF DNA polymerase (Finnzymes Ltd.). Amplification of the RNA/DNA hybrid was not successful (lane 1) but removal of the template RNA by RNase digestion yielded a clearly visible DNA product (lane 3). The DNase (lane 2) and DNase/RNase (lane 4) digested samples did not yield any amplification products. In order to determine the fidelity of reverse transcription, ssDNA gained following RNAse treatment was PCR amplified and the resulting DNA was sequenced. The quantity and quality of the resulting DNA sequence was compared to the activity of a commercial primer-dependent Moloney Murine Leukemia Virus (M-MLV) reverse transcriptase (Promega). The amount of dsDNA obtained after PCR amplification was comparable to the amount obtained by M-MLV reverse transcriptase. Throughout the sΔ^+^_13_ sequence, the VP1 RdRP and the M-MLV reverse transcriptase displayed two identical mismatches, both of which were attributed to the T7 RNA polymerase.
(TIFF)

Figure S6 **Birnavirus VP1 sequence alignment.** Selected representative birnavirus VP1 sequences are shown as follows (UniProt IDs in parentheses): IPNV, Infectious Pancreatic Necrosis virus strain Jasper (P22173); AY-98, marine birnavirus isolated from *Plecoglossus altivelis* (Q7T8V1); IBDV, Infectious Bursal Disease virus strain D78 (Q9Q6Q5); IBDV-L, Infectious Bursal Disease virus stain Lukert (Q49HH7); BSV, Blotched Snakehead virus (Q8AZL8); TV1, Tellina virus 1 (Q2PBR4); DSX, Drosophila X virus (Q91CD5). Numbering is for IPNV VP1 and moderately/highly conserved residues are shown in white type face with grey/black backgrounds, respectively. Note the conservation of residues that interact with the K^+^ ion (N184, N409 and N514), the only exceptions being the conservative substitution N514D in Drosophila X virus [42] and the substitution N514S in the severely-attenuated Lukert strain of IBDV [43]. Residues of the F5 binding pocket (W563, L578, R582 and F622) are strictly conserved except for the conservative substitution F662L in AY-98.
(PDF)

Figure S7 **The N-terminus of IPNV VP1 lies close to the (deoxy)nucleotide entrance tunnel.** The structure of VP1 is shown as a molecular surface for the well-folded polymerase domain (residues 28–792, white) and a cartoon for the N-terminal tail (orange). GTP molecules (spheres) modeled into the active site based on a structural superposition of the Φ6 RdRP initiation complex (PDB ID 1HI0) can be seen through the VP1 (deoxy)nucleotide entrance tunnel, revealing the proximity of the N-terminal tail to the exterior opening of this tunnel.
(TIFF)

Table S1 **Data collection and refinement statistics.** Numbers in parentheses refer to the appropriate outer shell.
(PDF)

Table S2 **RNA templates used in this study.**
(PDF)

Text S1 **Supplementary Materials and Methods.** ****
(PDF)
