# Supplementary figures and images for: The N-Terminus of the RNA Polymerase from Infectious Pancreatic Necrosis Virus Is the Determinant of Genome Attachment
Source: PLoS Pathog. 2011 Jun 23;7(6):e1002085. doi: 10.1371/journal.ppat.1002085 (PMC3121795; doi:10.1371/journal.ppat.1002085)

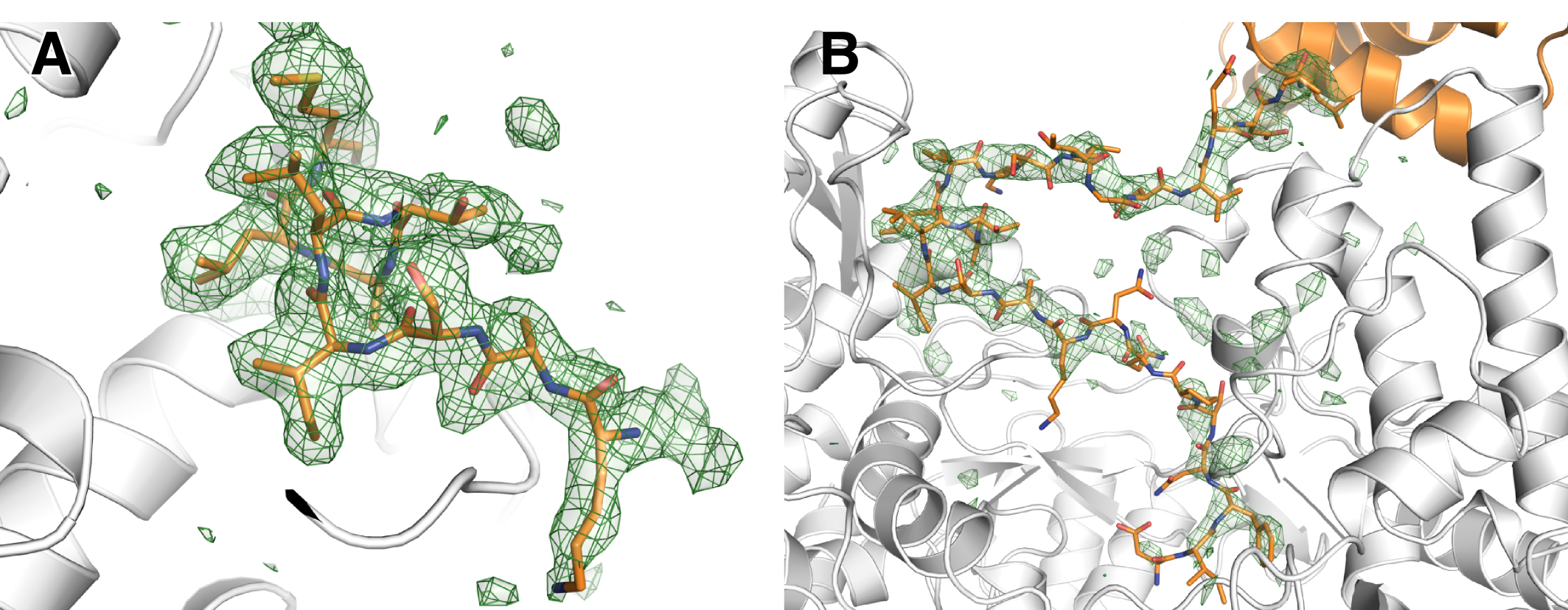

Supplement: Figure S1 — Binding of N-terminal tail to the VP1 active site cleft. The N-terminal tail bound in the active site clefts of (A) Mg-bound ΔC55 VP1, and (B) ΔC55 VP1 (‘large unit cell’). Unbiased F O-F C electron density, calculated before the N-terminal tail was added to the models, is shown in green (3.0 σ). The final refined structures of the polymerase domain and N-terminal tail are shown as a cartoon (white) and sticks (carbon atoms orange), respectively. In (B), the polymerase domain from which the N-terminal tail arises is shown as a cartoon (orange). (TIFF) [file ppat.1002085.s001.tiff]

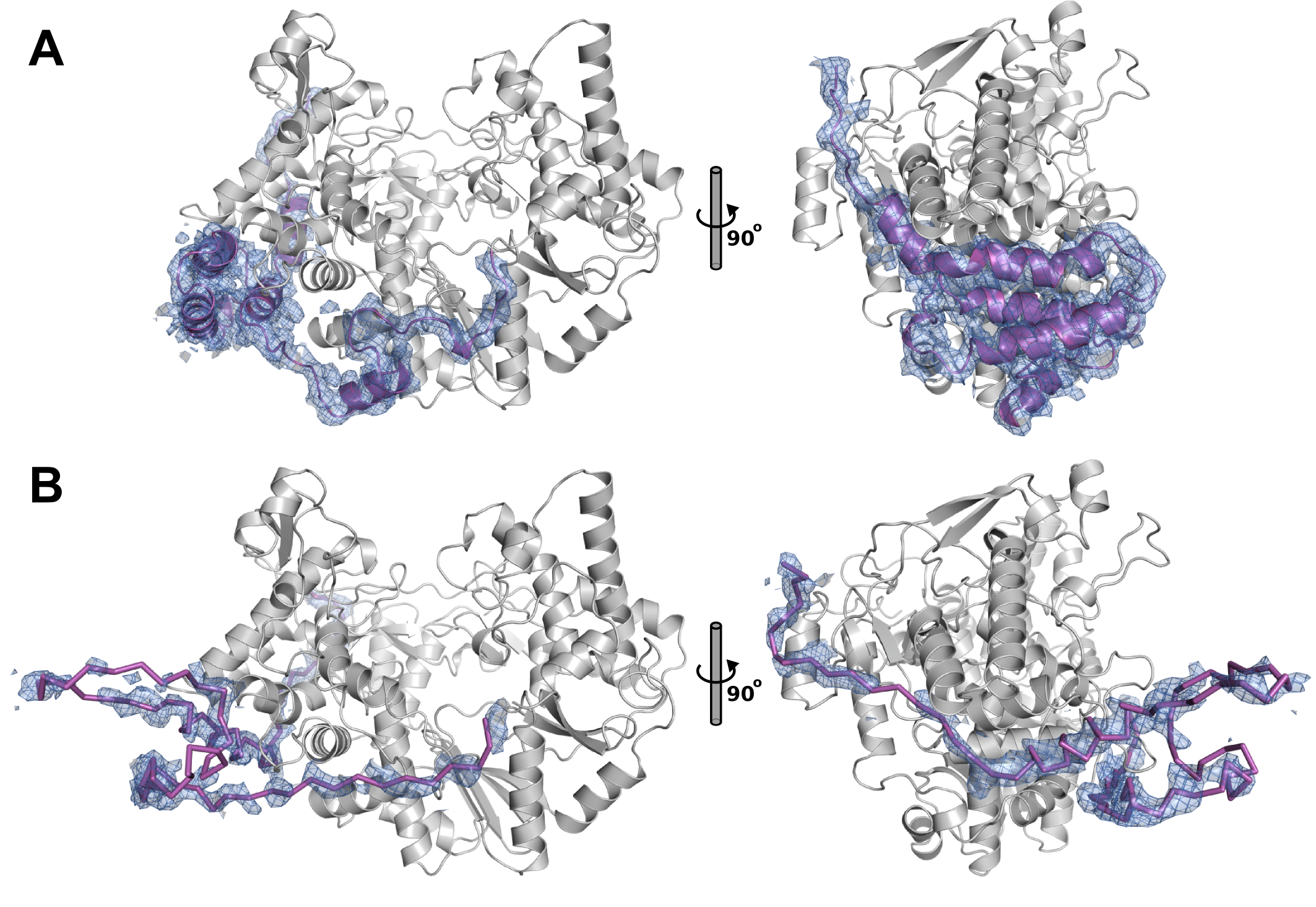

Supplement: Figure S2 — Reorganization of the C-terminal extension of full-length VP1. (A) Full-length VP1 with the C-terminal extension in the commonly observed conformation (chains A–C) is shown as a white (residues 3–687) and magenta (residues 688–799) cartoon. 2F O-F C electron density calculated from the final refined model is shown in blue (1.0 σ); for clarity density is shown only within a 2 Å radius of residues 688–799. (B) Full-length VP1 where the C-terminal extension is refolded (chain D) is shown as a white cartoon (residues 3–687) and a magenta Cα trace (residues 688 onwards). Electron density is as in (A), shown within a 3 Å radius of the Cα trace. Given the low resolution of the diffraction data we were unable to reliably build a main chain into the density and dock the sequence from residue 688 onwards in chain D. We have therefore excluded this region of the structure from the refinement, the deposited coordinates for this segment having zero occupancy to indicate this fact. (TIFF) [file ppat.1002085.s002.tiff]

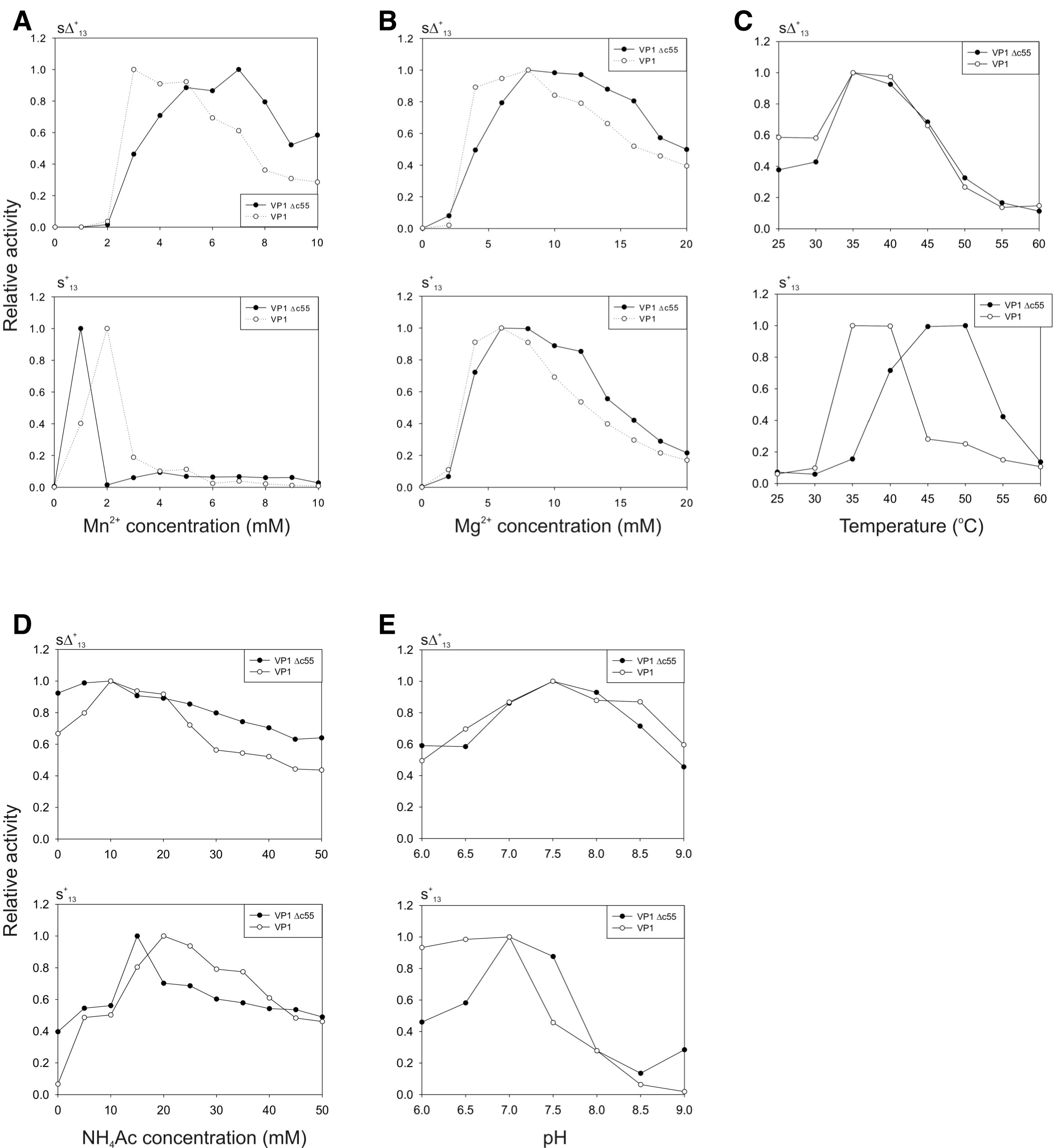

Supplement: Figure S3 — Biochemical characterization of IPNV VP1. Screening the optimal reaction conditions for the IPNV VP1 in terms of (A) Mn2+ concentration, (B) Mg2+ concentration, (C) temperature, (D) ammonium acetate (NH4Ac) concentration, and (E) pH. Results are normalized against the highest attained polymerization activity for each RdRP in each experiment. Two ssRNA templates of different lengths (sΔ+ 13, 723 nt, and s+ 13, 2961 nt) were used. Interestingly the optimal temperature for RNA polymerization and final RNA yield obtained depends on the length of the ssRNA template. VP1 efficiently processes the short template (sΔ+ 13) at 37°C, whereas when a long template (s+ 13) is applied the RdRP is most active at 45–50°C. Higher reaction temperatures lead to increased degradation of the ssRNA template and a subsequent drop in RNA synthesis. The product yield with short ssRNA templates is much higher than with long, although in all cases the yield is sufficiently high to be detected by ethidium bromide (EtBr) staining following agarose gel electrophoresis (not shown). (TIFF) [file ppat.1002085.s003.tiff]

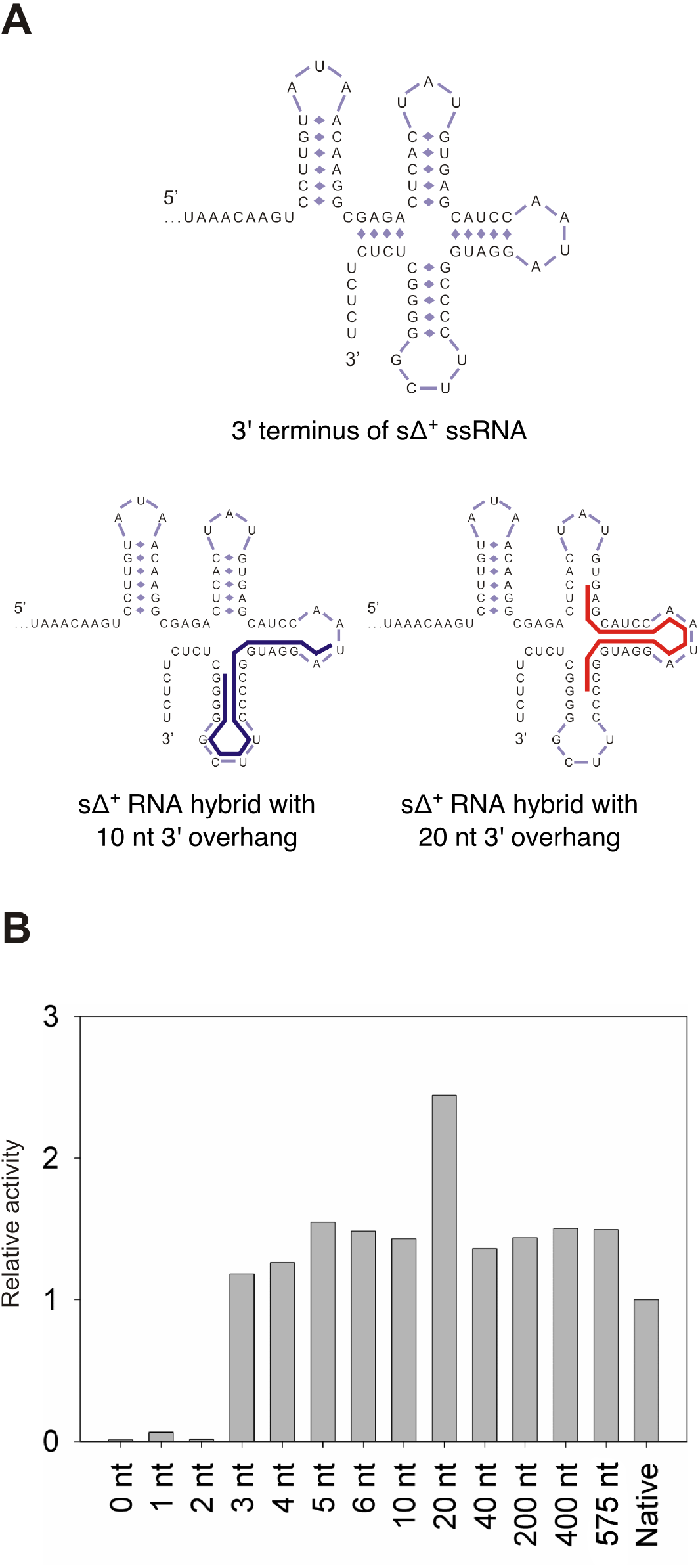

Supplement: Figure S4 — Oligonucleotide displacement assay. (A) Schematic representation of the secondary structure at the 3′ terminus of sΔ+ (upper panel) and of partially double-stranded sΔ+ RNA hybrids with 10 nucleotide (nt) and 20 nt 3′ overhangs (lower panels). The blue and red lines (lower panel) indicate the position at which the complementary RNA oligonucleotide anneals. (B) The RNA synthesis activity of full-length VP1 using sΔ+ ssRNA hybridized to short RNA oligonucleotides in order to produce RNA hybrids with different 3′ overhangs (0–575 nt) as template. The results have been normalized against unhybridized ssRNA sΔ+ 13 template (Native). The pre-initiation behavior of the VP1 polymerase is similar to that described for the Φ6 RdRP [41]. As with the Φ6 polymerase, RNA hybrids with single-stranded 3′ terminal overhangs of 0–2 nt induce very little RNA synthesis, whereas efficient RNA synthesis occurs using templates with 3′ overhangs of 3 nt or longer. Interestingly, the RNA hybrid with a 20 nt 3′ overhang stimulates approximately 2.5-fold higher RNA production activity than the unhybridized ssRNA sΔ+ 13 template. (TIFF) [file ppat.1002085.s004.tiff]

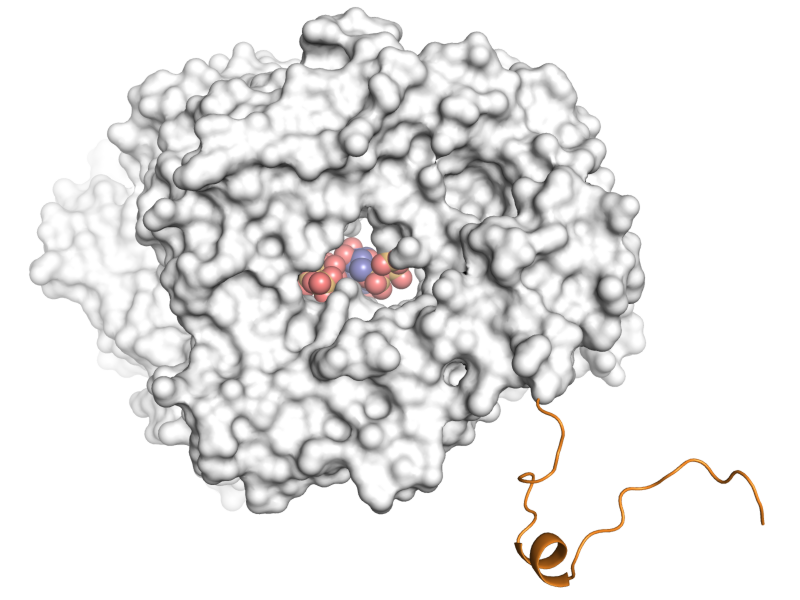

Supplement: Figure S7 — The N-terminus of IPNV VP1 lies close to the (deoxy)nucleotide entrance tunnel. The structure of VP1 is shown as a molecular surface for the well-folded polymerase domain (residues 28–792, white) and a cartoon for the N-terminal tail (orange). GTP molecules (spheres) modeled into the active site based on a structural superposition of the Φ6 RdRP initiation complex (PDB ID 1HI0) can be seen through the VP1 (deoxy)nucleotide entrance tunnel, revealing the proximity of the N-terminal tail to the exterior opening of this tunnel. (TIFF) [file ppat.1002085.s007.tiff]
