# Supplementary material for: The N-Terminus of the RNA Polymerase from Infectious Pancreatic Necrosis Virus Is the Determinant of Genome Attachment
Source: PLoS Pathog. 2011 Jun 23;7(6):e1002085. doi: 10.1371/journal.ppat.1002085 (PMC3121795; doi:10.1371/journal.ppat.1002085)
Supplement: Table S1 — Data collection and refinement statistics. Numbers in parentheses refer to the appropriate outer shell. (PDF) [file ppat.1002085.s008.pdf]

	apo ΔC55 VP1	Mg-bound ΔC55 VP1	*apo* ΔC55 VP1 (large unit cell)	Full-length VP1
**Data collection**				
Beamline	Diamond I02	Diamond I02	Diamond I02	ESRF ID14-2
Wavelength (Å)	0.9793	0.9793	0.9800	0.9330
Resolution limits (Å)	92.1–2.3 (2.36–2.30)	101.8–2.2 (2.26–2.20)	145.0–3.0 (3.10–3.02)	45.2–3.8 (4.01–3.80)
Space group	$P2_12_12_1$	$P2_12_12_1$	$P2_12_12_1$	$P2_12_12_1$
Unit cell dimensions (Å)	a = 134.3, b = 184.3, c = 244.9	a = 134.1, b = 183.9, c = 244.5	a = 200.9, b = 209.5, c = 243.4	a = 119.7, b = 195.5, c = 197.4
Unique reflections	268,352 (19,741)	304,685 (22,296)	200,159 (14,668)	45,508 (6,612)
Redundancy	7.2 (7.2)	7.3 (7.4)	4.3 (4.4)	4.9 (4.7)
Completeness (%)	100.0 (100.0)	100.0 (100.0)	100.0 (100.0)	98.5 (99.0)
$I/\sigma(I)$	10.3 (2.9)	10.6 (2.7)	8.6 (2.0)	4.5 (1.8)
R_{merge} (%)[a]	0.207 (0.732)	0.166 (0.752)	0.156 (0.858)	0.300 (0.815)
R_{meas} (%)[b]	0.222 (0.788)	0.179 (0.809)	0.178 (0.977)	0.336 (0.917)
R_{pim} (%)[c]	0.082 (0.291)	0.066 (0.297)	0.085 (0.464)	0.148 (0.410)
Mosaicity (°)	0.33	0.28	0.20	1.06
**Refinement**				
Resolution limits (Å)	90.3–2.3 (2.36–2.30)	32.2–2.2 (2.26–2.20)	145.0–3.0 (3.10–3.02)	44.4–3.8 (3.90–3.80)
Number of reflections in working set	264,144 (19,348)	301,493 (22,029)	198,026 (13,888)	43,166 (3,181)
Number of reflections in test set	2,655 (190)	3,070 (246)	2,029 (145)	2,307 (169)
R_{xpct} (%)[d]	0.166 (0.195)	0.161 (0.191)	0.188 (0.249)	0.186 (0.219)
R_{free} (%)[e]	0.187 (0.213)	0.181 (0.222)	0.216 (0.270)	0.213 (0.251)
Number of molecules per asymmetric unit	5	5	8	4
Number of atoms (protein/water/other[f])	30,154/3,143/11	30,143/2,989/16	49,229/0/8	23,768/0/0
Number of atoms with alternate conformations (protein/water/other[f])	163/0/0	122/0/0	0/–/0	0/–/–
Residues in Ramachandran favored region (%)	97.9	98.0	97.4	97.5
Ramachandran outliers (%)	0.0	0.0	0.1	0.1
r.m.s.d bond lengths (Å)[g]	0.011	0.011	0.011	0.009
r.m.s.d bond angles (°)[g]	1.03	0.98	1.13	1.08
Wilson B (Å^2)	23.3	28.0	51.0	83.8
Average B factors (Å^2) (protein/water/other[f])	17.2/26.2/14.4	26.9/35.6/25.4	65.1/–/83.8	99.5/–/–

[a]$R_{merge} = \Sigma_{hkl} \Sigma_i |I(hkl;i) - \langle I(hkl)\rangle| / \Sigma_{hkl} \Sigma_i I(hkl;i)$, where $I(hkl;i)$ is the intensity of an individual measurement of a reflection and $\langle I(hkl)\rangle$ is the average intensity of that reflection

[b]R_{meas} is the redundancy-independent merging R factor [Diederichs K, Karplus PA (1997) Improved R-factors for diffraction data analysis in macromolecular crystallography. Nat Struct Biol 4: 269-275.]

[c]R_{pim} is the precision-indicating merging *R* factor [Weiss MS, Hilgenfeld R (1997) On the use of the merging R factor as a quality indicator for X-ray data. Journal of Applied Crystallography 30: 203-205; Weiss MS, Metzner HJ, Hilgenfeld R (1998) Two non-proline cis peptide bonds may be important for factor XIII function. FEBS Lett 423: 291-296.]

[d]$R_{xpct} = \Sigma_{hkl}||F_{obs}| - |F_{xpct}||/\Sigma_{hkl} |F_{obs}|$, where $|F_{obs}|$ and $|F_{xpct}|$ are the observed structure factor amplitude and the expectation of the model structure factor amplitude, respectively

[e]R_{free} equals R_{xpct} of the test set (1–5% of the data removed prior to refinement)

[f]Other includes magnesium, potassium and chloride ions

[g]r.m.s.d. is root mean square deviation from ideal geometry
