# Supplementary material for: The N-Terminus of the RNA Polymerase from Infectious Pancreatic Necrosis Virus Is the Determinant of Genome Attachment
Source: PLoS Pathog. 2011 Jun 23;7(6):e1002085. doi: 10.1371/journal.ppat.1002085 (PMC3121795; doi:10.1371/journal.ppat.1002085)
Supplement: Table S2 — RNA templates used in this study. (PDF) [file ppat.1002085.s009.pdf]

Template:	Description:	Type:	Size:	Reference:
ϕ6 dsRNA	Bacteriophage ϕ6 large genome segment (L-segment)	dsRNA	6 374 bp	[1]
	Bacteriophage ϕ6 medium genome segment (M-segment)	dsRNA	4 063 bp	[1]
	Bacteriophage ϕ6 small genome segment (S-segment)	dsRNA	2 948 bp	[1]
s^{+}_{rep}	Positive-sense transcript of PCR amplified fragment corresponding to the last 300 bp of bacteriophage ϕ6 S-segment	ssRNA	300 nt	[2]
$s\Delta^{+}_{A}$	Positive-sense transcript of bacteriophage ϕ6 S-segment with an internal deletion (bases 593-2838); contains an additional A at the 3'-end	ssRNA	711 nt	[3]
$s\Delta^{+}_{C}$	Positive-sense transcript of bacteriophage ϕ6 S-segment with an internal deletion (bases 593-2838); contains an additional C at the 3'-end	ssRNA	711 nt	[3]
$s\Delta^{+}_{G}$	Positive-sense transcript of bacteriophage ϕ6 S-segment with an internal deletion (bases 593-2838); contains an additional G at the 3'-end	ssRNA	711 nt	[3]
$s\Delta^{+}_{U}$	Positive-sense transcript of bacteriophage ϕ6 S-segment with an internal deletion (bases 593-2838); contains an additional U at the 3'-end	ssRNA	711 nt	[3]
$s\Delta^{+}_{13}$	Positive-sense transcript of bacteriophage ϕ6 S-segment with an internal deletion (bases 593-2838); contains the …CUAGAGGAUCCCC addition at the 3'-end	ssRNA	723 nt	[4]
$s\Delta^{+}_{HP}$	Positive-sense transcript of bacteriophage ϕ6 S-segment with an internal deletion (bases 593-2838); contains the …CUAGGGGUUCGCCCC addition that forms a stable tetra-loop at the 3'-end	ssRNA	725 nt	[4]
s^{+}_{13}	Positive-sense transcript of bacteriophage ϕ6 S-segment with the …CUAGAGGAUCCCC addition at the 3'-end	ssRNA	2 961 nt	[5]

1. Bamford DH, Ojala PM, Frilander M, Walin L, Bamford JKH (1995) Isolation, purification, and function of assembly intermediates and subviral particles of bacteriophages PRD1 and Φ6. In: Adolph KW, editor. Methods in molecular genetics. San Diego: Academic Press. pp. 455-474.
2. Sarin LP, Poranen MM, Lehti NM, Ravantti JJ, Koivunen MRL, et al. (2009) Insights into the pre-initiation events of bacteriophage Φ6 RNA-dependent RNA polymerase: towards the assembly of a productive binary complex. Nucleic Acids Res 37: 1182-1192.

3. Makeyev EV, Bamford DH (2000) The polymerase subunit of a dsRNA virus plays a central role in the regulation of viral RNA metabolism. EMBO J 19: 6275-6284.
4. Laurila MR, Makeyev EV, Bamford DH (2002) Bacteriophage Φ6 RNA-dependent RNA polymerase: molecular details of initiating nucleic acid synthesis without primer. J Biol Chem 277: 17117-17124.
5. Gottlieb P, Strassman J, Qiao X, Frilander M, Frucht A, et al. (1992) In vitro packaging and replication of individual genomic segments of bacteriophage Φ6 RNA. J Virol 66: 2611-2616.
