# Supplementary material for: The N-Terminus of the RNA Polymerase from Infectious Pancreatic Necrosis Virus Is the Determinant of Genome Attachment
Source: PLoS Pathog. 2011 Jun 23;7(6):e1002085. doi: 10.1371/journal.ppat.1002085 (PMC3121795; doi:10.1371/journal.ppat.1002085)
Supplement: Text S1 — Supplementary Materials and Methods. (PDF) [file ppat.1002085.s010.pdf]

Supplementary Materials and Methods

Cloning ΔC55 and ΔN27C55 VP1

A shortened VP1 construct lacking the C-terminal 55 residues (ΔC55) was designed based on PONDR disorder predictions [1] and secondary structure analysis [2]. ΔC55 VP1 was amplified from the plasmid encoding full-length VP1 using KOD Hot Start DNA polymerase (Novagen) according to the manufacturer's instructions (sense primer: 5′-ggggacaagtttgtacaaaaaagcaggcttcgaaggagatagaaccatgtcggacatcttcaactcaccac-3′; antisense primer: 5′-ggggaccactttgtacaagaaagctgggtctcaatggtgatggtgatgatgaccggtcttctccagtgcttctcttgtctccgtcag-3′). The forward and reverse primers appended sequences encoding a start codon and a C-terminal $KTGH_6$ tag to the start and end of the clone, respectively, and both primers contained the attB site of the Gateway cloning system (Invitrogen). The PCR product was sub-cloned into the expression vector pDEST14 (Invitrogen). VP1 lacking both the C-terminal 55 and N-terminal 27 residues (ΔN27C55 VP1) was amplified from the plasmid encoding full-length VP1 using KOD DNA Polymerase (Novagen) according to the manufacturers instructions (sense primer: 5′-aggagatataccatggacgtgctgataccaaaacgcttc-3′; antisense primer: 5′-gtgatggtgatgtttctccagtgcttctcttgtctccg-3′) and cloned into pOPINF, encoding a C-terminal KH_6 tag, by ligation-independent cloning [3].

Expression and Purification of IPNV VP1

Full-length, ΔC55, ΔN27C55 and mutant VP1 were expressed in Rosetta(DE3)pLysS cells and overexpression was initiated by autoinduction [4]. Cultures were grown at 37°C to an A_{595} of 0.6–0.8 (4–6 h), cooled to 25°C and allowed to grow for a further 16–20 h before harvesting by centrifugation (8000g at 8°C for 10 min). Cell pellets were stored at -80°C until required.

Frozen bacterial pellets were thawed and resuspended on ice in 50 mM Tris, 500 mM NaCl, 50 mM imidazole (pH 7.5) supplemented with protease inhibitors (Roche or Sigma) and 400 units of bovine pancreas deoxyribonuclease I (Sigma) per 1 L of cell culture. Cells were lysed using a Basic Z model cell disruptor (Constant Systems) and lysate was cleared by centrifugation (35,000g at 8°C for 30 min). Cleared lysate was applied to a 1 mL HisTrap Ni immobilisation affinity column (GE Healthcare), the column was washed with ten column volumes of 50 mM Tris, 500 mM NaCl, 50 mM imidazole (pH 7.5), eluted in 50 mM Tris, 500 mM NaCl, 500 mM imidazole (pH 7.5) and the eluate immediately applied to a HiLoad 16/60 Superdex 200 column (GE Healthcare) equilibrated in 20 mM Tris, 200 mM mM NaCl (pH 7.5). Fractions containing pure VP1 were pooled and concentrated in 10 kDa MWCO centrifugal concentrators (Millipore).

For crystallization of full-length VP1, the C-terminal His_6 affinity tag was removed by incubation with 100 µL of carboxypeptidase A conjugated to agarose beads (Sigma) in 20 mM Tris pH 7.8 and 300 mM NaCl overnight at room temperature. The cleaved protein was separated from the beads by centrifugation (8,000g, 5 min) and applied to 1 mL of Ni-agarose beads (GE Healthcare), the flow-through being pooled and re-purified by gel-filtration as described above.

Production of RNA templates

Synthetic ssRNA was produced by run-off transcription *in vitro* with T7 RNA polymerase [5], templates for the transcription being prepared by either cutting the plasmid DNA with restriction endonucleases or by PCR amplification. RNA templates $s\Delta^{+}_{13}$ (ssRNA, 723 nt) and $s\Delta^{+}_{HP}$ (ssRNA, 723 nt) were transcribed from *Sma*I digested plasmid pEM15 and pEM19, respectively [6], and s^{+}_{TTT} (ssRNA, 1928 nt) and s^{+}_{13} (ssRNA, 2961 nt) were transcribed from *Bst*BI- and *Sma*I-digested plasmid pLM659 [7], respectively. The template-specificity RNAs

($s\Delta^{+}$, $s\Delta^{+}_{A}$, $s\Delta^{+}_{C}$, $s\Delta^{+}_{G}$, and $s\Delta^{+}_{U}$, all 711 nt) were synthesized from DNA following PCR amplification of pEM15 using DyNAzyme II polymerase (Finnzymes) and oligonucleotides 1–11 (see table below) [6,8]. The template s^{+}_{rep} was transcribed from the 300 bp PCR amplified fragment of plasmid pLM659 (oligonucleotides 2 and 12, see table below). Genomic dsRNA substrates (L: 6374 bp; M: 4063 bp; S: 2948 bp) were obtained from full-length Φ6 bacteriophage infections [9]. All RNAs were dissolved in sterile 18 Ω milli-Q water (Millipore Corporation) and the concentrations were determined by spectrophotometry (A_{260}). The quality of each preparation was monitored by electrophoresis in 1% w/v agarose (TBE) gels [10]. For an overview of the RNA templates please refer to Table S2.

	**Oligonucleotide**	**Sequence (5′ – 3′)**	**Description / Reference**
1.	T7-1	CGCGTAATACGACTCACTATAG	Upstream [5]
2.	3’end	AGAGAGAGAGCCCCCGA	Downstream [8]
3.	3’end_1	AAGAGAGAGAGCCCCCGA	Downstream [8]
4.	3’end_2	CAGAGAGAGAGCCCCCGA	Downstream [8]
5.	3’end_3	GAGAGAGAGAGCCCCCGA	Downstream [8]
6.	3’end_4	TAGAGAGAGAGCCCCCGA	Downstream [8]
7.	pT7_3'end	TAAGCTTGGGCTGCAGGT	Downstream [5]
8.	pT7_3'end_1	ATAAGCTTGGGCTGCAGGT	Downstream [8]
9.	pT7_3'end_2	CTAAGCTTGGGCTGCAGGT	Downstream [8]
10.	pT7_3'end_3	GTAAGCTTGGGCTGCAGGT	Downstream [8]
11.	pT7_3'end_4	TTAAGCTTGGGCTGCAGGT	Downstream [8]
12.	T7-Phi6_2628-2650_fwd	CGCGTAATACGAACTATAGGGAC-ACGGAAGTTATTGAAGCAGC	Upstream [11]

Supplementary Materials and Methods References

1. Romero P, Obradovic Z, Li X, Garner EC, Brown CJ, et al. (2001) Sequence complexity of disordered protein. Proteins 42: 38-48.

2. Cole C, Barber JD, Barton GJ (2008) The Jpred 3 secondary structure prediction server. Nucleic Acids Res 36: W197-201.

3. Berrow NS, Alderton D, Sainsbury S, Nettleship J, Assenberg R, et al. (2007) A versatile ligation-independent cloning method suitable for high-throughput expression screening applications. Nucleic Acids Res 35: e45.

4. Studier FW (2005) Protein production by auto-induction in high density shaking cultures. Protein Expr Purif 41: 207-234.

5. Makeyev EV, Bamford DH (2000) Replicase activity of purified recombinant protein P2 of double-stranded RNA bacteriophage Φ6. EMBO J 19: 124-133.

6. Makeyev EV, Bamford DH (2000) The polymerase subunit of a dsRNA virus plays a central role in the regulation of viral RNA metabolism. EMBO J 19: 6275-6284.

7. Gottlieb P, Strassman J, Qiao X, Frilander M, Frucht A, et al. (1992) In vitro packaging and replication of individual genomic segments of bacteriophage Φ6 RNA. J Virol 66: 2611-2616.

8. Makeyev EV, Bamford DH (2001) Primer-independent RNA sequencing with bacteriophage Φ6 RNA polymerase and chain terminators. RNA 7: 774-781.

9. Bamford DH, Ojala PM, Frilander M, Walin L, Bamford JKH (1995) Isolation, purification, and function of assembly intermediates and subviral particles of bacteriophages PRD1 and Φ6. In: Adolph KW, editor. Methods in molecular genetics. San Diego: Academic Press. pp. 455-474.

10. Sambrook J, Russell D (2001) Molecular cloning: a laboratory manual (3rd edition). Cold Spring Harbor, New York: Cold Spring Harbor Laboratory Press.

11. Sarin LP, Poranen MM, Lehti NM, Ravantti JJ, Koivunen MRL, et al. (2009) Insights into the pre-initiation events of bacteriophage Φ6 RNA-dependent RNA polymerase: towards the assembly of a productive binary complex. Nucleic Acids Res 37: 1182-1192.
